# Evaluation of Oral Mucositis Occurrence in Oncologic Patients under Antineoplastic Therapy Submitted to the Low-Level Laser Coadjuvant Therapy

**DOI:** 10.3390/jcm7050090

**Published:** 2018-04-24

**Authors:** Alessandro Leite Cavalcanti, Dário José de Macêdo, Fernanda Suely Barros Dantas, Karla dos Santos Menezes, Diego Filipe Bezerra Silva, William Alves de Melo Junior, Alidianne Fabia Cabral Cavalcanti

**Affiliations:** 1 Programa de Pós-Graduação em Saúde Pública, Universidade Estadual da Paraíba, Avenida das Baraúnas, S/N—Campus Universitário, Bodocongó, Campina Grande 58109-753, Brazil; 2 Faculty of Medical Sciences, Campina Grande 58109-753, Brazil; dario.jmacedo@gmail.com; 3 School of Dentistry, State University of Paraíba, Campina Grande 58109-753, Brazil; fernandasbdantas@gmail.com (F.S.B.D.); fernandasbdant@gmail.com (K.d.S.M.); fernandasbdant1s@gmail.com (D.F.B.S.); 4 Hospital Universitário Alcides Carneiro, Federal University of Campina Grande, Campina Grande 58109-753, Brazil; williamgeronto@gmail.com; 5 Department of Dentistry, State University of Paraiba, Campina Grande 58109-753, Brazil; alidianne.fabia@gmail.com

**Keywords:** low-level laser therapy, mucositis, antineoplastic agents

## Abstract

Low-level laser therapy has been widely used in treating many conditions, including oral mucositis. The purpose of this study was to evaluate the occurrence of oral mucositis in patients undergoing antineoplastic therapy submitted to preventive and therapeutic treatment with low-level laser therapy. This cross-sectional study was carried out with 51 children and adolescents of both sexes with malignant neoplasias who developed oral mucositis and underwent low-level laser therapy. Data were collected on sex, age, type and degree of neoplasia, region affected, and remission time. 64.7% of the patients were male and were between 3 and 6 years of age (39.2%). Acute lymphoid leukemia was the most frequent neoplasm (37.3%). Regarding the maximum oral mucositis, grade 2 (41.2%) was predominant, with jugal mucosa (29.9%) and tongue (17.7%) being the most affected regions. The majority of cases presented lesion remission time between 4 and 7 days (44.0%). Most patients were young, male, and diagnosed with acute lymphoid leukemia. Predominance of grade 2 oral mucositis was observed, with jugal mucosa and tongue being the most affected regions, with the majority of cases presenting lesion remission time between 4 and 7 days. Low-level laser therapy has been shown to be an essential therapy in the prevention and treatment of these lesions, since it is a non-invasive and low-cost method.

## 1. Introduction

The main forms of treatment for malignant neoplasms are chemotherapy and radiotherapy, which often cause various side effects such as oral mucositis (OM) [1,2,3,4,5,6,7,8,9,10]. This condition begins asymptomatic and, later, an erythema appears, which may be followed by erosion or ulceration of the oral mucosa, sometimes covered by a fibrinopurulent pseudomembrane and may lead to spontaneous bleeding [3,11].

With lesion evolution, there is an increased risk of local and systemic infections, pain, and compromised phonation and swallowing [1,4,12,13,14,15,16]. As a result of intense pain, patients are unable to orally feed and end up by being parenterally or venously fed [4,7,13]. Depending on the OM severity, the patient may have prolonged hospitalization and unplanned treatment interruptions, which may severely affect the results of antineoplastic therapy [7].

Several therapeutic approaches have been used to prevent and treat OM in all treatment modalities for malignant neoplasms [16]. Use of mouthwashes, analgesics, antibiotics, and anti-inflammatory drugs are used to prevent and treat OM [3].

Low-level laser therapy (LLLT) has been used both to prevent and treat OM induced by various antineoplastic therapies [3,17,18]. It consists of the specific application of a monochromatic light source of a narrow high-density spectrum with wavelength ranging from visible red to infrared [19]. It is believed that LLLT acts in a beneficial and non-invasive way [1], being also able to induce biological effects like analgesia [3] and aid in tissue repair through increased vascularization, cellular motility, and reepithelialization [14,16,18,19]. Nevertheless, in relation to its use for OM prevention, few studies with specific protocols have been conducted [17].

Thus, the purpose of the present study was to evaluate the occurrence of OM in patients under antineoplastic therapy submitted to preventive and therapeutic LLLT treatment.

## 2. Materials and Methods

### 2.1. Study Design

This cross-sectional study was developed in the Oncopediatrics sector of the *Alcides Carneiro* University Hospital (HUAC) in Campina Grande, Brazil.

### 2.2. Sampling

The population involved children and adolescents undergoing antineoplastic treatment from January 2014 to December 2015 and who developed oral mucositis (OM). Fifty-one patients aged 3–19 years, of both sexes, were admitted to the Oncopediatrics sector.

Information regarding sex, age, and diagnosis was collected from medical records. OM classification was performed according to classification proposed by the World Health Organization (WHO, Geneva, Switzerland) [20], according to Table 1. Data regarding the OM grade, lesion location and remission, and laser therapy protocols were also collected.

All patients were submitted to the LLLT protocol [19], which consisted of low-level laser applications with 3.3 J/cm^2^ of irradiation intensity, spot size 3 mm^2^, and variable wavelength, according to the OM grade. Patients with LLLT prescriptions before or during chemotherapy and with grade 0 OM received daily laser applications with wavelengths of 660 nm (nanometers) for 10 s at several marked points of the oral mucosa (right and left jugal oral mucosa (five points on each side), 5 points on the palate, 3 points on the lower lip, 1 point on the upper lip, 3 points on the back of the tongue, 3 points on the lateral borders of the tongue and 3 points on the oral floor), which may vary according to size of the patient’s oral cavity, so as to cover the entire oral region. In the case of patients with OM grade 1 or higher, the same procedure described previously was applied in addition to applications with wavelength of 808 nm for 10 s on the OM surface.

MMO Laser Duo Portable (MMOptics Ltda., São Carlos, Brazil) device was used, with the following technical specifications: GaAlAs and InGaAlP type emitter semiconductor laser, continuous frequency mode, power of 100 mW, wavelength of 660 nm or 808 nm, and exit point of 3 mm^2^.

### 2.3. Statistical Analysis

Data were organized in Excel^®^ for Windows (version 2013, Excel, Microsoft Inc., Redmond, WA, USA) spreadsheet and submitted to statistical analysis with SPSS (IBM Inc., Chicago, IL, USA).

### 2.4. Ethical Aspects

This study was approved by the Research Ethics Board of the Cruzeiro do Sul University, Protocol No. 176/2014.

## 3. Results

The demographic and clinical characteristics of patients are shown in Table 2. The majority were male (64.7%) aged 3–6 years at the start of treatment (39.1%). Regarding diagnosis, acute lymphocytic leukemia (ALL) accounted for 37.3% of cases. Regarding maximum OM grade, the majority (41.2%) was classified as grade 2.

As to the affected site, there was predominance of jugal mucosa (29.9%) and tongue (17.7%) (Table 3).

The majority of cases presented lesion remission between 4 and 7 days (44.0%), while 18% of patients had no lesion recurrence (Table 4).

## 4. Discussion

The cytotoxic effect of chemotherapy on the basal layer of epithelial cells leads to a decrease in the renewal rate of these cells, with atrophy and tissue ulceration [1], which characterizes oral mucositis. This condition is the result of several etiological agents and has characteristics that change as it progresses [1]. In response to primary damage (message generation phase), a number of transcription factors are activated and there is production of proinflammatory cytokines [5,6], nitric oxide, ceramide, and matrix metalloproteinases (MMPs), leading to apoptosis and tissue injury. Inflammatory modulators are activated and promote signal amplification that drives the destructive process, so that the oral epithelium eventually disintegrates and ulcerates (ulceration phase) [6].

After the ulceration phase, the healing phase occurs. Acceleration of wound healing occurs through increased release of growth factors, increased neovascularization, and collagen formation [5]. These biological events of oral mucositis are influenced by several factors such as toxicity, dosage, patient’s general health, susceptibility to chemotherapeutic agents, dental conditions, and oral hygiene [1,21].

Chemotherapy-induced oral mucositis on non-keratinized mucosa is usually manifested during the first and second weeks of chemotherapy sessions [4]. The ulcerated oral epithelium allows the entry of microorganisms into the oral cavity, which can cause local and systemic infections [1]. Due to oral pain, patients tend to become dehydrated and malnourished [1,6,16].

Oral mucositis occurs in approximately 23.8% [22] to 82% [21] of children receiving antineoplastic treatment, and those with haematological malignancies present OM more frequently than those with solid tumors [13]. This result was observed in the present study, since the majority of patients had acute lymphoid leukemia (37.3%) and acute myeloid leukemia (13.7%). Brazilian authors showed that individuals with severe difficulty in transporting oneself were more susceptible to developing oral mucositis [22].

Age has shown to be a risk factor for oral mucositis, with younger individuals presenting this condition more frequently and with higher grades, probably due to the high cell renewal rate [23,24,25]. The majority of participants in this study were children between 3 and 6 years of age (39.1%).

There was predominance of grade 2 OM, similar to that reported by other authors [8,21]. Tongue, soft palate, oral floor, and oral mucosa are the regions most affected by OM [12]. In this research, the jugal mucosa and the tongue were the anatomic regions most affected by oral mucositis.

One of the therapeutic methods used to prevent or treat oral mucositis is low-level laser therapy, since it has the capacity to induce various biological effects, such as analgesia and modulation of the inflammatory process [3,14].

The laser acts on cellular enzymes that increase the mechanism of the oxidative chain in mitochondria (cell potency), which results in an increase in the production of adenosine triphosphate (ATP) [5,7], producing intracellular reactive oxygen species [5]. Different wavelengths act at different tissue levels. In particular, light from 632 to 660 nm works on superficial layers and in epithelial tissues, and from 780 to 901 nm, light penetrates much deeper into subepithelial tissues [16]. These wavelengths are similar to the LLLT protocols used in this study.

Much research has shown the beneficial effects of LLLT on the treatment of oral mucositis [4,21,26], being therefore a prophylactic treatment more effective than treatment after symptoms appear [6].

Most of the lesions showed remission within 7 days or had no recurrence, which proves the efficacy of LLLT in the prevention and treatment of OM and its importance as an additional adjuvant method in the routine of patients receiving antineoplastic treatment.

Cancer has a negative influence on the quality of life of children and adolescents with the disease [27,28]. The inclusion of dentists in the oncology staff is of paramount importance for patient care at all stages of disease. Cancer patients require specific oral care and should be monitored before, during, and after anticancer treatment to minimize the deleterious effects of chemotherapy and improve their quality of life [29,30,31].

Given the particular vulnerability exhibited by children undergoing anticancer treatment, protocols are highly relevant. Based on the complications associated with anticancer treatment, the establishment of educational, preventive, and curative strategies targeting oral health before, during, and after treatment aimed at the resolution and/or minimization of discomfort of patients and their relatives has paramount importance [26].

## 5. Conclusions

Most patients were young, male, and diagnosed with ALL. There was a predominance of grade 2 oral mucositis, and jugal mucosa and tongue were the most affected regions, with the majority of cases presenting lesion remission between 4 and 7 days. Therefore, LLLT has been shown to be an essential therapy for these lesions, since it is a non-invasive and low-cost method.

## Figures and Tables

**Table 1:** OM classification [20].

Score	0	1	2	3	4
Signals and symptoms	No change	Erythema or pain	Erythema, ulcer; can feed on solids	Ulcers; requires strictly liquid diet	Oral feeding is not possible

**Table 2:** Distribution of oncopediatric patients according to demographic and clinical characteristics.

Characteristics	*n* (%)
Sex	
Male	33 (64.7)
Female	18 (35.3)
Age (years)	
3–6	20 (39.21)
7–12	14 (27.45)
13–19	17 (33.33)
Diagnostic	
Acute lymphocytic leukemia (ALL)	19 (37.3)
Acute myeloid leukemia (AML)	7 (13.7)
Hodgkin’s Lymphoma	3 (5.9)
Neuroblastoma	3 (5.9)
Rhabdomyosarcoma	3 (5.9)
Non-Hodgkin’s Lymphoma	2 (3.9)
Medulloblastoma	2 (3.9)
Histiocytosis	2 (3.9)
Lymphoblastic lymphoma T	1 (2.0)
Juvenile myelomonocytic leukemia	1 (2.0)
T cell lymphoid leukemia	1 (2.0)
Lumbosacral column adenocarcinoma	1 (2.0)
Neuroblastoma	1 (2.0)
Undifferentiated sarcoma	1 (2.0)
Primary lymphoma of the central nervous system	1 (2.0)
Burkitt’s lymphoma	1 (2.0)
Ewing’s Sarcoma	1 (2.0)
Lymphoblastic lymphoma	1 (2.0)
Maximum grade of Oral Mucositis	
0	10 (19.6)
1	3 (5.9)
2	21 (41.2)
3	12 (23.5)
4	4 (7.8)
No information	1 (2.0)

**Table 3:** Distribution of patients according to the OM sites.

Anatomical Location	*n* (%)
Jugal mucosa	38 (29.9)
Tongue	20 (17.7)
Lower lip	18 (14.2)
Palate	15 (11.8)
Upper lip	12 (9.5)
Gum	10 (7.9)
Lingual frenulum	7 (5.5)
Comissuras	5 (3.9)
Lip frenulum	2 (1.6)
BASE ^1^	127 (100.0)

^1^ The patient could present the disease in more than one anatomical region.

**Table 4:** Period of oral mucositis remission.

Lesions Recurrence (*n* = 50)	*n* (%)
4–7 days	22 (44.0)
8–12 days	10 (20.0)
13–19 days	9 (18.0)
No recurrence observed	9 (18.0)
